# Fission Yeast Nod1 Is a Component of Cortical Nodes Involved in Cell Size Control and Division Site Placement

**DOI:** 10.1371/journal.pone.0054142

**Published:** 2013-01-17

**Authors:** Isabelle Jourdain, Elspeth A. Brzezińska, Takashi Toda

**Affiliations:** Cell Regulation Laboratory, London Research Institute, Cancer Research UK, London, United Kingdom; Newcastle University, United Kingdom

## Abstract

Most cells enter mitosis once they have reached a defined size. In the fission yeast *Schizosaccharomyces pombe*, mitotic entry is orchestrated by a geometry-sensing mechanism that involves the Cdk1/Cdc2-inhibiting Wee1 kinase. The factors upstream of Wee1 gather together in interphase to form a characteristic medial and cortical belt of nodes. Nodes are also considered to be precursors of the cytokinesis contractile actomyosin ring (CAR). Here we describe a new component of the interphase nodes and cytokinesis rings, which we named Nod1. Consistent with its role in cell size control at division, *nod1Δ* cells were elongated and epistatic with regulators of Wee1. Through biochemical and localisation studies, we placed Nod1 in a complex with the Rho-guanine nucleotide exchange factor Gef2. Nod1 and Gef2 mutually recruited each other in nodes and Nod1 also assembles Gef2 in rings. Like *gef2Δ*, *nod1Δ* cells showed a mild displacement of their division plane and this phenotype was severely exacerbated when the parallel Polo kinase pathway was also compromised. We conclude that Nod1 specifies the division site by localising Gef2 to the mitotic cell middle. Previous work showed that Gef2 in turn anchors factors that control the spatio-temporal recruitment of the actin nucleation machinery. It is believed that the actin filaments originated from the nodes pull nodes together into a single contractile ring. Surprisingly however, we found that node proteins could form pre-ring helical filaments in a *cdc12-112* mutant in which nucleation of the actin ring is impaired. Furthermore, the deletion of either *nod1* or *gef2* created an un-expected situation where different ring components were recruited sequentially rather than simultaneously. At later stages of cytokinesis, these various rings appeared inter-fitted rather than merged. This study brings a new slant to the understanding of CAR assembly and function.

## Introduction

The fission yeast *Schizosaccharomyces pombe* constitutes an excellent model organism in which to study the mechanisms that control cell size. Fission yeast is rod shaped and grows by tip extension along its long axis. When it reaches its critical size, *S. pombe* enters mitosis and divides by equatorial fission, yielding two daughter cells of equal length. The transition between growth and division occurs in G2/M and is under the control of the cyclin-dependent kinase Cdk1/Cdc2. In short interphase cells, Cdk1 is kept inactive by Wee1-dependent phosphorylation of tyrosine 15. In longer cells, Cdk1 is de-inhibited by Cdc25-dependent de-phosphorylation of this residue and subsequently activates a myriad of substrates that coordinate cell cycle progression through M phase [1], [2].

**Figure 1:**
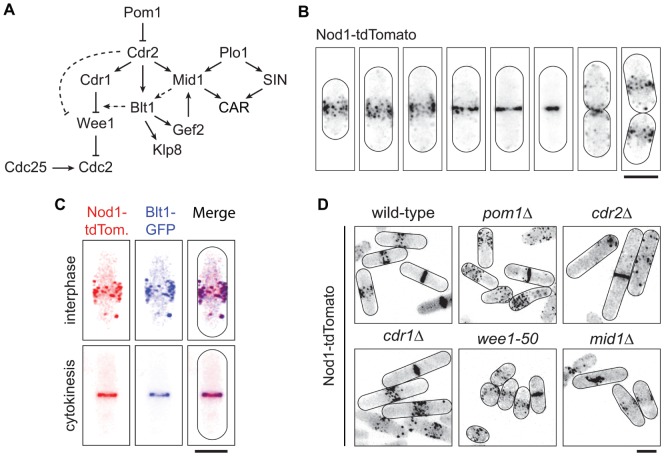
Nod1 belongs to the interphasic broad band of nodes. (A) Nodes pathway. Interphase node proteins control Cdc2 phosphorylation and mitotic entry. See text for details. (B) Nod1-tdTomato localisation during the *S. pombe* cell cycle. In interphase, Nod1 localises to a cortical belt of nodes, which congress into cytokinesis nodes first and then into a contractile ring. (C) Nod1-tdTomato co-localises with Blt1-GFP in nodes (top panel) and rings (bottom panel). (D) Nod1-tdTomato was expressed in various mutants involved in the nodes pathway. The localisation of Nod1 in nodes is altered in mutants of the most upstream regulators Pom1 and Cdr2. Nod1 is always present in the cytokinetic ring. Bars = 5 µm.

In the vegetative cell cycle, fission yeast cells either grow (G1-S-G2 phases) or divide (M phase) and cells that cannot divide keep growing. A delayed mitotic entry, equivalent to a longer stay in G2, generates long cells, whereas a premature entry into mitosis produces cells that divide at a short length. The timing of division is therefore critical for the definition of cell size in fission yeast. A number of recent studies have linked mitotic progression to a novel cell geometry-sensing mechanism [3], [4]. The factors involved appear to be upstream regulators of Wee1. They organise as an equatorial, cortical broad band of nodes (aka midsome) that overlay the nucleus in interphase. Known components of interphase nodes include the kinase Cdr2, the kinase Nim1/Cdr1, the Rho-Gef (Guanine nucleotide Exchange Factor) Gef2, the kinesin-like Klp8, a protein of unknown function Blt1 and in a smaller amount, the kinase Wee1 and the anillin Mid1 [5]–[8]. Cdr2 is the master organiser of the belt, which gathers nodes at the medial cell cortex. Gradients of proteins such as the Pom1 kinase, emanating from the cell poles, prevent Cdr2 from accumulating at the cell tips and therefore control the medial localisation of the interphase nodes. The deletion of each component of the nodes leads to a cell length phenotype, due to the delayed (long cells, e.g. *cdr2Δ*) or premature (short cells, e.g. *wee1* mutant) entry in mitosis. The current model proposes that cell length at division is determined by the distance separating the medial belt of nodes from the cell tips [8], [9]. When cells are short, the polar gradient of Pom1 reaches the cell middle, where it triggers a cascade of inhibitory phosphorylations leading to Wee1 activation, Cdk1/Cdc2 inhibition and mitotic delay. As cells elongate, the Pom1 gradient weakens in the cell mid-zone and can no longer phosphorylate Cdr2 [8]. De-inhibited Cdr2 inactivates Wee1 [10]–[12], and induces progression through M phase (see Figure 1A for a current view).

Besides sensing cell geometry, the nodes are also involved in the spatio-temporal assembly of the cytokinetic ring. At the onset of mitosis, the interphase nodes condense into cytokinesis nodes that are considered to be the precursors of the contractile actomyosin ring (CAR) [13]. Mid1 rather than Cdr2, is central to this process. Upon mitotic entry, the nuclear pool of Mid1 is signalled into the nodes where it is activated to interact with proteins involved in CAR architecture and function [14], [15]. In the “search, capture and pull model”, Mid1 recruits cytoskeletal proteins such as the formin Cdc12 or the type II myosin (heavy chain Myo2 and light chains Cdc4 and Rlc1). Cdc12 nucleates short actin filaments from individual nodes [16]–[19]. While elongating in random directions, these actin filaments are captured by myosins loaded on neighbouring nodes. Myosins generate the force necessary to pull the nodes together and, along with actin bundling proteins, complete the construction of the CAR [20]–[22]. In the absence of Mid1 function, rings are assembled by a parallel pathway (Septation Initiation Network, SIN, [23], [24]), but are mis-oriented or offset [18], [25], [26]. The nodes and SIN pathways that control CAR assembly are both under the upstream regulation of the Polo kinase Plo1 (Figure 1A) [14], [27].

Different subsets of midsome proteins are present at different stages of the nodes/ring cycles. Cdr2 disappears from the nodes as they condense into a ring and Mid1 returns back to the nucleus at the onset of ring constriction [5], [6]. By contrast, other interphase node components, such as Gef2 and Blt1, remain in the cytokinesis nodes and are present in the CAR until the end of cytokinesis [8]. An interaction map within the nodes and the rings is gradually emerging but the function of each protein in either cell size control or ring functionality remains largely unknown.

Here we report the identification of a new component of the nodes, which we named Nod1. We show that Nod1 forms a complex with Gef2 downstream of Blt1, and recruits Gef2 to both nodes and rings. We also made the surprising discovery that different nodes components form inter-fitted rings, that assemble, constrict and disassemble independently.

## Materials and Methods

### Yeast Genetics, Culture and Strains

Media, growth, genetics and maintenance of strains were as described in [28]. Cells were cultured to mid-log phase in YE5S or EMM at 27°C.

Yeast strains used in this study are described in Supplementary Table S1. Deletion strains and carboxy-epitope-tagged proteins were generated *via* chromosomal integration of PCR-amplified fragments [29], [30]. The presence of the tag did not affect any of the proteins used in this study.

### Cell Imaging and Fluorescence Microscopy

Live cell imaging was performed in an imaging chamber (CoverWell 20-mm diameter 0.5-mm deep; Molecular Probes) filled with 800 µl of 2% agarose in EMM and sealed with a 22×22-mm glass coverslip. Cells were imaged using an Olympus IX71 wide-field inverted epifluorescence microscope with the Deltavision-SoftWoRx system (Olympus and Applied Precision Co.), in a temperature controlled environmental chamber. Olympus UPlanSapo 63x or 100x NA 1.4, oil immersion objective was used and images captured with a Coolsnap-HQ digital CCD camera or a Cascade EMCCD 512B camera (Roper Scientific). Pictures of Z-sections were deconvolved and projected. Counts, measurements and image presentations were made using Metamorph (Molecular Devices Corporation) and downloaded to Microsoft Excel or Prism for analysis. For all node data, the localisations described concerned virtually all cells and were therefore not scored, but images are representative of >300 cells observed in at least two independent experiments. Similarly, pictures of reconstructed rings are representative of most, if not all rings.

Cell length at division was measured on cells stained with calcofluor. A stock solution of calcofluor white (fluorescent brightener 28 Sigma) was prepared at 5 mg/ml in H_2_O, vortexed for up to 24 h and centrifuged to eliminate the undissolved powder. One μλ of this suspension was pipetted onto cells pre-applied to an agarose pad, and a coverslip was sealed. Cells were observed immediately.

### Co-Immunoprecipitation

Cells were lysed in extraction buffer (50 mM Hepes 50 mM NaF 50 mM Na-β-glycerophosphate 5 mM EGTA 5 mM EDTA 0.2% Triton X100 1x Protease Inhibitor Cocktail 1 mM PMSF) by the mechanical action of acid-washed glass beads (FastPrep FP120 apparatus, Savant. Co., 2×25 s power 5.5). Debris was eliminated by centrifugation for 1 min, then 5 min at 13000 g at 4°C. Protein concentrations were determined by Bradford assay (Biorad). For co-immunoprecipitations, and unless stated otherwise, 0.5–4 mg of whole cell extract (WCE) was incubated for 2 h at 4°C with protein-A Dynabeads coated with antibodies against 3PK (mouse monoclonal Serotec), Flag (rabbit polyclonal, Sigma), or GFP (rabbit polyclonal, Invitrogen). Beads were then extensively washed (50 mM Tris-HCl pH = 7.4 1 mM EDTA 150 mM NaCl 0.05% NP-40 10% Glycerol 1 mM DTT 1.5 mM PNPP 1×Protease Inhibitor Cocktail 0.1 mM PMSF) and boiled for 5 min in Laemmli sample buffer. Forty µg of whole cell extract (WCE) was similarly treated and used as controls. Proteins were separated on denaturing 4–12% gradient gels (BioRad) transferred onto PVDF membranes which were further blocked and immune-blotted in the presence of 10% non-fat milk. Primary antibodies against 3PK (mouse monoclonal, Serotec), Flag (M2 mouse monoclonal, Sigma) or GFP (mouse monoclonal, Roche) were used diluted to 1∶1000 in ImmunoShot Solution 1 (2B Scientific). For secondary antibodies, anti-mouse TrueBlot Ultra HRP-conjugated (eBioscience) was used at a dilution of 1∶2000 in ImmunoShot Solution 2 (2B Scientific). Signals were detected using ECL (GE Healthcare).

## Results

We previously identified SPAC12B10.10 open reading frame (ORF) as a new cell cycle regulator [31]. SPAC12B10.10 is a non essential gene whose 419 amino acid product is predicted to contain a C-terminus coiled-coil domain. As the protein localises to cortical nodes (Figure 1B), we decided to name it Nod1, and hypothesised that it may have a role in cell size control at division (see below).

### Nod1 is Part of the Machinery that Controls Cell Size at Division

To characterise Nod1 in fission yeast, we chromosomally tagged it at the C-terminus with fluorescent proteins (GFP or tdTomato). The protein was not affected by the presence of the tags, as judged by normal cell growth and morphology in any medium and at any temperature (unpublished results). In the wild-type background, Nod1 was expressed throughout the cell cycle. In interphase, it localised to a belt of cortical dots at the cell middle (Figure 1B). These structures remained equatorial from G1 to G2 and showed little movement. At the onset of mitosis, the Nod1 nodes condensed into a contractile ring and later re-appeared at the equator of each interphasic daughter cell. This localisation was reminiscent of that of node proteins such as Blt1. Figure 1C confirms that the two proteins indeed perfectly co-localised, both in interphase and at cytokinesis.

As node-containing proteins are normally involved in the control of mitotic entry, we asked if the absence of *nod1* would affect cell size. Indeed, *nod1Δ* cells divided at a slightly, yet significant, longer size than the wild-type (Table 1). Mitotic entry is controlled by the antagonising effect on Cdc2 activation of Wee1 kinase and Cdc25 phosphatase. To see where Nod1 acts, we tested for genetic interactions between *nod1* and genes involved in either pathway (Figure 1A). The length phenotype of *nod1Δ* and *cdc25–22* cells was additive, indicating that both genes lie in parallel pathways (Table 1). By contrast, *cdr2*, which encodes an upstream regulator of Wee1, was epistatic to *nod1*. Indeed, *nod1Δcdr2Δ* cells divided at the same increased size as the *cdr2Δ* single mutant, indicating that Nod1 and Cdr2 act in the same pathway for G2/M transition control. Hence, Nod1 is a new member of the cortical belt of nodes and controls cell size through Wee1.

**Table 1:** Cell length at division of indicated strains grown in rich (YE5S) or minimum (EMM) media at 27°C.

Strain	YE5S	EMM
wild-type	14.1±0.2 (n = 302)	12.38±0.02 (n = 246)
*nod1Δ*	14.9±0.5 (n = 331)	13.38±0.29 (n = 238)
*cdc25–22*	ND	20.61±0.03 (n = 237)
*nod1Δ cdc25–22*	ND	24.90±0.16 (n = 184)
*cdr2Δ*	17.9±0.5 (n = 194)	18.43±0.61 (n = 183)
*nod1Δ cdr2Δ*	17.5±0.1 (n = 384)	18.92±0.17 (n = 201)

Mean±SD µm, n cells measured, *p*<0.005.

### Nod1 Interacts with Gef2, Downstream of Blt1

**Figure 2:**
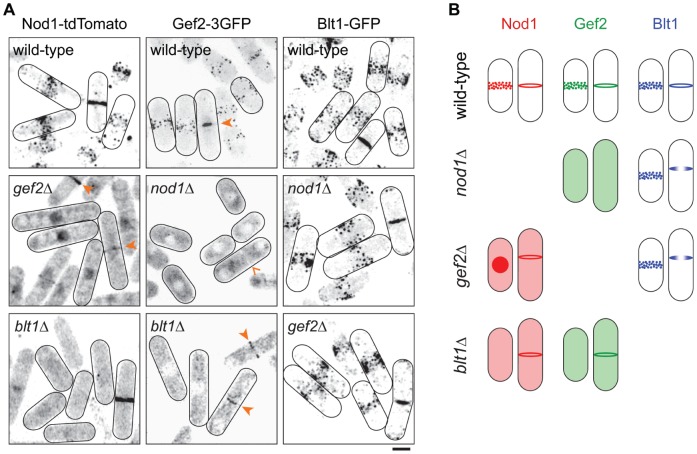
Nod1, Gef2 and Blt1 depend on one another for their localisation. (A) Localisation of Nod1-tdTomato, Gef2–3GFP and Blt1-GFP in wild-type, *nod1Δ*, *gef2Δ* or *blt1Δ* cells. Plain arrowheads point at cytokinetic rings. The open arrowhead in Gef2-3GFP *nod1Δ* (middle row), shows a cytokinetic cell with two separated nuclei, where the cytokinetic ring is not stained by Gef2-3GFP. Bar = 5 µm. (B) Schematic summary of cells shown in (A). Localisation patterns of Nod1-tdTomato (red), Gef2-3GFP (green) and Blt1-GFP (blue) are depicted. The ring phenotype observed in *gef2Δ* and *nod1Δ*cells is described in Figure 4.

To place Nod1 in the nodes pathway, we observed its localisation in mutants of the pathway. Like all node proteins, the concentration of Nod1 in the medial belt was dependent on Pom1 [8], [9], [32], [33]. As expected, in *pom1Δ* cells, Nod1 was spread in one half of the cells, towards the non-growing tip (Figure 1D). Nod1 nodes were also delocalised from the medial cortex in *cdr2Δ* cells and the signal intensity of Nod1-tdTomato in nodes was present but reduced in *mid1Δ* cells [7], [8]. Nod1 is probably not part of the Cdr1-Wee1 branch of the pathway, as its localisation was unaffected in *cdr1Δ* and *wee1–50* cells (Figure 1D). By contrast, Nod1 was totally absent from the nodes in the *blt1Δ* and *gef2Δ* strains (Figure 2A). In both cases Nod1 was cytoplasmic but in the absence of *gef2* it was also nuclear and visibly chromatin-associated. No mis-localisation was observed in a strain lacking *klp8*, a downstream effector of Blt1 (data not shown). In all the strains described above, Nod1 was present in rings, regardless of their positioning or orientation.

We next examined the localisation-dependency of Nod1, Blt1 and Gef2 relative to one another (Figure 2A). In wild-type cells, all three proteins were observed throughout the cell cycle, in medial cortical nodes and in cytokinetic rings. Similar to Nod1-tdTomato and as described previously [7], in the absence of *blt1*, Gef2-3GFP disappeared from the nodes, but not the rings. By contrast, Blt1-GFP localisation was unaffected by the deletion of either *nod1* or *gef2*. Nod1 and Gef2 depended on each other for their localisation in nodes. The recruitment of Gef2–3GFP in rings was also compromised in the *nod1Δ* strain. These findings that are summarised in Figure 2B suggest that in terms of nodes localisation, Blt1 is upstream of Nod1 and Gef2.

**Figure 3:**
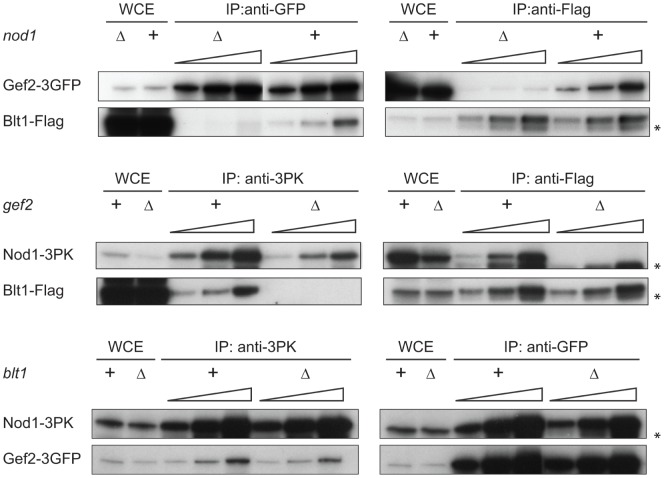
Nod1, Gef2 and Blt1 depend on one another for their interaction. Co-immunoprecipitations of Nod1-3PK, Gef2-3GFP and Blt1-Flag in the presence (+) or absence (Δ) of *nod1*, *gef2* or *blt1*. To compensate for the decreased levels of proteins in the mutants compared to the wild-type, increasing amounts of whole cell extracts were loaded on the gels (1x, 2x and 4x). WCE: Whole Cell Extract (40 µg). Asterisk: unspecific band. All 3 proteins interact with one another. Nod1 helps the Gef2-Blt1 interaction; Gef2 is required for the Nod1-Blt1 interaction; Blt1 however does not affect the Nod1-Gef2 interaction.

We further investigated if these three proteins could physically interact with one another. For this purpose, we created strains in which Nod1 was chromosomally tagged with 3PK, Blt1 with 3Flag and Gef2 with 3GFP. We first noticed that Nod1-3PK was less immunodetected in whole cell extracts prepared from strains lacking *blt1* and *gef2*, than in the wild-type or any other mutant of the nodes pathway (Supplementary Figure S1). This suggested that the stability of Nod1 depends on Blt1 and Gef2. Reciprocal co-immunoprecipitations further showed that in wild-type cells, Nod1, Blt1 and Gef2 interact with one another (Figure 3, “+”). To see if the three proteins depended on one another for their mutual interaction, we systematically observed the interaction between two of them in the absence of the third one (Figure 3, “Δ”). The absence of *nod1* strongly affected the Blt1-Gef2 interaction (Figure 3, top panel) and the absence of *gef2* abrogated the Blt1-Nod1 interaction (Figure 3, middle panel). By contrast, Nod1 and Gef2 co-immunopreciptated equally well in the presence or absence of *blt1* (Figure 3, bottom panel). Together, these results suggest that Nod1 forms a complex with Gef2, capable of interacting with Blt1.

### The Absence of Gef2 or Nod1 Reveals that Node Proteins can be Recruited Separately and Form Independent Rings

**Figure 4:**
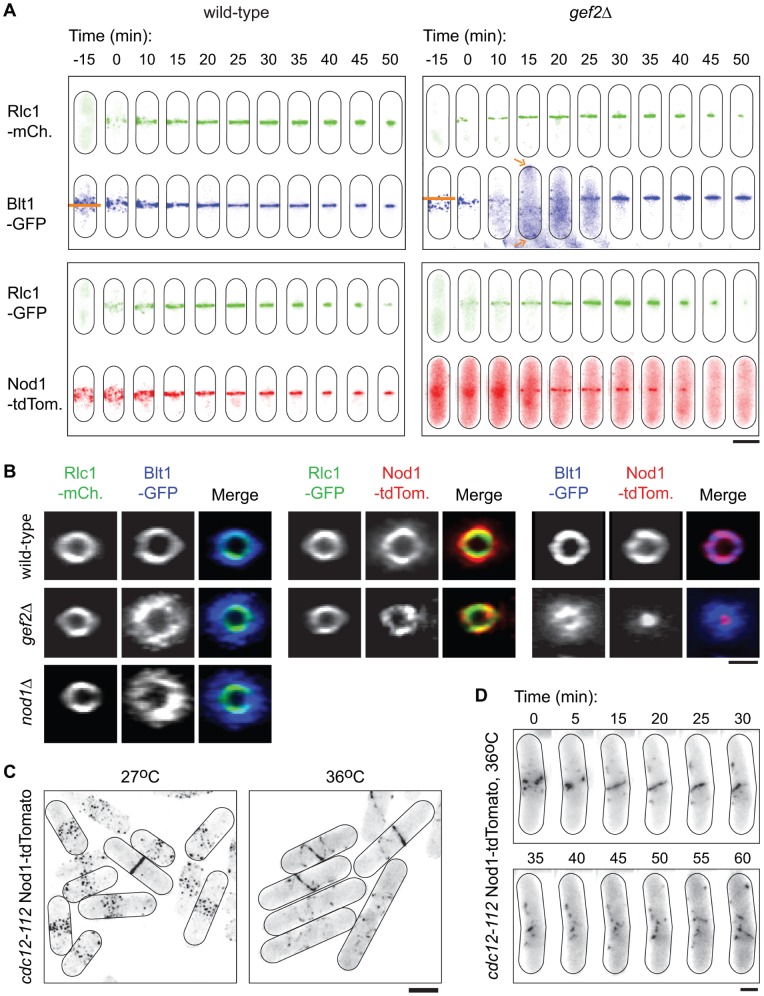
The absence of Gef2 reveals the existence of independently assembling and contracting cytokinesis rings. (A) Time lapse images of cells expressing Blt1-GFP and Rlc1-mCherry (top panel) or Nod1-tdTomato and Rlc1-GFP (bottom panel) in whole wild-type (left panel) and *gef2Δ* cells (right panel) during cytokinesis. Time 0 corresponds to the first recruitment of Rlc1 in cytokinesis nodes. Orange horizontal lines mark the site of future ring assembly, in the middle of the belt of nodes in the wild-type, and at its edge in *gef2Δ*. Orange arrows point at the cell tips localisation of Blt1-GFP in the *gef2Δ* cell (see also Supplementary Figure S2). (B) Transverse views of the midzone of cells expressing the indicated combinations of tagged proteins. Note the inter-fitted rings in the absence of *gef2* or *nod1*. Images are representative of 2-8 reconstructed rings but this pattern is seen in virtually all cytokinetic cells. (C) Nod1-tdTomato in *cdc12-112* cells incubated at 27°C or 36°C for one generation. (D) Time lapse images of Nod1-tdTomato in a representative *cdc12-112* cell observed at the restrictive temperature. Nodes first fused into a long filament (t = 0 to 20 min), which later fragmented (t = 35 to 50 min), and fused again towards the end of the time lapse (t = 55 to 60 min). Bar in (A and C) = 5 µm; bars in (B and D) = 2 µm.

Gef2 was recently shown to regulate contractile ring stability at the end of cytokinesis [7]. To assess if this function involved its partners Nod1 and Blt1, we followed their localisation through cytokinesis in the presence or absence of *gef2*. As a read out of actin ring progression, we used the cytokinetic myosin light chain Rlc1, tagged with either –GFP or –mCherry (Figure 4A). In wild-type cells, Nod1-tdTomato and Blt1-GFP co-localised in interphase nodes and throughout their condensation in cytokinesis nodes. Projected views of whole cells showed that the signals also perfectly overlapped during ring formation, constriction and dissolution. Rlc1 was not present in interphase nodes, but coincided with Nod1 and Blt1 from its first appearance in cytokinesis nodes and until the end of CAR constriction (Figure 4A, left panel). In *gef2Δ*, Rlc1 also appeared in cytokinesis nodes. Nod1-tdTomato remained cytoplasmic and nuclear during this stage and only joined the actomyosin ring once it was fully assembled (Figure 4A, right panel). The Rlc1-labelled actin ring and the Nod1-containing ring then constricted alongside each other. Blt1-GFP was pre-established in the interphase nodes. Concomitant with the gradual appearance of Rlc1 in cytokinesis nodes, Blt1-GFP was inversely expulsed from them and translocated to the cell tips (arrows in the top panel in Figure 4A). A few minutes after Nod1 was seen as a ring and immediately prior to the start of constriction, Blt1-GFP came back to the cell middle and overlapped with the other rings (See also Supplementary Figure S2). Thus, in wild-type conditions, Nod1, Blt1 and Rlc1 simultaneously organise as a ring, whereas in *gef2Δ* cells, Rlc1 is first recruited to the ring, followed by Nod1 and finally Blt1.

Intriguingly, abnormal ring organisation in *gef2Δ* cells extended to the later stages of cytokinesis. Firstly, the Blt1 ring did not constrict. Instead, the signal spread as a single disk in a manner similar to the closing primary septum. However, the calcofluor-stained primary septum marked the outer border of the Blt1-GFP disk and the two structures did not appear to co-localise (Supplementary Figure S3A). The closed Blt1 disk remained at the interface between the two daughter cells until the end of cytokinesis, long after Nod1 and Rlc1 had vanished. Secondly, towards the end of cytokinesis, the Nod1 signal looked unevenly distributed, as if the Nod1 ring had collapsed. In fact, at the end of ring constriction, the Rlc1, Nod1 and Blt1 rings that overlapped in wild-type cells, appeared imbricated in *gef2Δ* cells (Figure 4B).

Since Gef2 localisation in rings is Nod1-dependent (see above, Figure 2), we hypothesised that abnormal rings would be observed also in the absence of Nod1. As expected, Blt1-GFP behaved in an identical manner in *nod1Δ* and in *gef2Δ* cells (Supplementary Figure S2). This result confirms that Nod1 and Gef2 work together, not only in interphase but also in cytokinesis.

### Nod1 and Blt1 can form Long Filaments in the Absence of an Actin Ring

The data presented above (Figure 4A and B) suggested that the function of Nod1 and Blt1 is not to recruit the actomyosin machinery to the cytokinetic ring. As we found a condition in which Nod1 and Blt1 were recruited after Rlc1, we wondered if the actin ring was, on the contrary, essential for the recruitment of Nod1 and Blt1. To answer this question, we observed the localisation of Nod1, Blt1 and Rlc1 in the *cdc12-112* temperature sensitive strain. Cdc12 is the only cytokinesis-specific actin-nucleating formin in *S. pombe* [34]. We confirmed that contrarily to wild-type cells, *cdc12-112* cells are incapable of organising an Rlc1-containing actomyosin ring at the restrictive temperature (Supplementary Figure S3B). Surprisingly however, we found that Nod1-tdTomato organised into short fragments (41.6%±1.7, n = 237) or longer filaments (33.0%±0.0, n = 237) that formed by the fusion of existing, medial and cortical nodes. The longer filaments were more easily observed when they spread across the full length of the cells, appearing as spring-like structures (Figure 4C). Filaments assembled and disassembled through cycles of fusions/fragmentations, supposedly in failed attempts of the cells to initiate cytokinesis (Figure 4D). Blt1-GFP was similarly capable of forming these elongated filaments (Supplementary Figure S3B). We were unfortunately unable to observe Gef2-3GFP in these cells because its signal faded away at elevated temperatures, even in wild-type conditions. We conclude that Nod1 and Blt1 can form presumptive pre-ring filamentous fibres, independently of actin.

### Nod1 Helps Define the Position of the Division Plane

**Figure 5:**
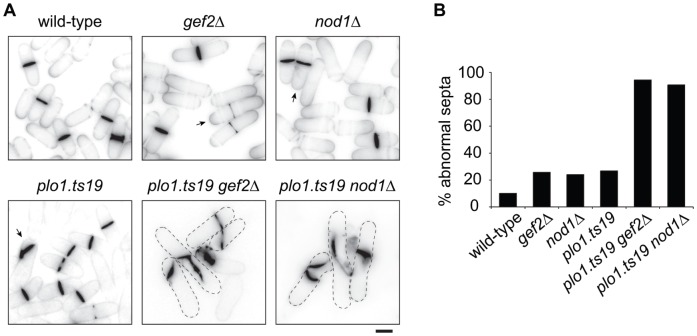
Nod1 is involved in the positioning of the division plane. (A) Calcofluor-labelled septa in the indicated *plo1* ^+^ (top) and *plo1-ts19* (bottom) strains. Arrows show examples of abnormal septa in *gef2Δ*, *nod1Δ* and *plo1.ts19* single mutants. Bar = 5 µm. (B) Percentage of cells with abnormal septa in the indicated strains. In *gef2Δ*, *nod1Δ* and *plo1.ts19* cells, this percentage is representative of offset septa, whereas double mutants show a more extensive septation phenotype (eg. multiple septa, oblique septum, etc).

In wild-type cells, the cytokinetic ring forms at a position that previously was the middle of the belt of nodes. We noticed that in the *gef2Δ* mutant, the Blt1-GFP ring formed at the edge of the belt, i.e. slightly offset (Supplementary Movie S1). Consequently, approximately 25% of *gef2Δ* cells, showed an asymmetrical division (wild-type = 10.1%±3.5; *gef2Δ* = 24.1%±5.7, Figure 5A and B). As we found that Gef2 is recruited to the ring by Nod1 (see above, Figure 2), we suspected that Nod1 also had a function in ring placement. As expected, approx. 25% of *nod1Δ* cells showed a slight shift of their division plane towards one tip (*nod1Δ* = 25.7%±4.7). This finding suggested a role of Gef2 and Nod1 in the positioning of the division plane. This is in good agreement with a report from Ye et al [7] who found that a defect in division site positioning is best seen in *gef2Δ* cells when the parallel placement pathway controlled by the Polo kinase Plo1 is also affected [27], [35]. Similar to the situation in *gef2Δ plo1.ts19* mutants, virtually all *nod1Δ plo1.ts19* cells formed aberrant septa, which were dramatically offset, tilted and eventually supernumerary (Figure 5A and B). By contrast, septa in *nod1Δ* and mutant alleles of *plo1* were far less affected. Thus, through the recruitment of Gef2, Nod1 plays a role in the specification of the division site.

## Discussion

### Blt1 Recruits the Nod1-Gef2 Complex to Interphase Nodes

Fission yeast cells spend most of their cell cycle in G2 interphase. The immunoprecipitation data that we described were obtained from asynchronous cultures and likely concern interactions that occur in interphase nodes. Accordingly, the interaction dependencies matched the localisation dependencies in interphase nodes. For example, in cells deleted of *nod1*, the Blt1-Gef2 interaction was no longer detected, and Blt1 was still observed in nodes whereas Gef2 was not. Similarly, in the absence of *gef2*, Blt1 and Nod1 no longer interacted and their localisation in nodes was uncoupled. In other words, when either *nod1* or *gef2* was deleted, Blt1 was still observed in the nodes. Moreover, when Nod1 or Gef2 was delocalised from the nodes (in *gef2Δ* or *nod1Δ* strains), Blt1 was also visible in the cortical belt. Hence, the localisation of Blt1 is independent of both the expression and localisation of Nod1 and Gef2. However, Nod1 and Gef2 are recruited to the nodes in a manner dependent on Blt1. The fact that the absence of *blt1* does not alter the formation of the Nod1-Gef2 complex but does modify the localisation of both in the nodes, suggests that Blt1 recruits Nod1 and Gef2 as a complex. This interaction will likely find its significance with the discovery of the GTPase that Gef2 activates.

### Nod1 Recruits Gef2 in Cytokinesis and Helps Position the Division Plane

Nod1, Gef2 and Blt1 are the only three proteins of the Wee1 pathway to be expressed both in the nodes and rings, and this must be of significance for cell division. Because Blt1 sits upstream of Nod1 and Gef2 in interphase, we initially thought that it may also have a similar regulatory role during cytokinesis. However, Blt1 recruits Nod1 and Gef2 to interphase nodes, but not cytokinesis rings ([7] and this study). Moreover, we were unable to detect any defect in ring(s), placement and recruitment in the absence of *blt1* [15]. Finally, the *blt1Δ* strain does not show a synthetic phenotype when combined with the *plo1* mutant [7]. Whereas these evidences do not permit to conclude on the role of Blt1 during cytokinesis, it was recently proposed that Blt1 acts in parallel with Mid1 to stably anchor cytokinetic nodes to the cell cortex and act as a scaffold for ring precursors [36]. In this model, Gef2 may be recruited by yet other factors to mediate the Blt1-Mid1 interaction.

We show here that Nod1 is one of the factors that recruit Gef2 to the cell equator not only in interphase but also in cytokinesis. The 145 C-terminal amino-acids of Gef2 are responsible for its localisation in all stages of the cell cycle [7], and although we have not investigated this, it is likely that this region of Gef2 mediates its interaction with Nod1. Along with Plo1, Gef2 was shown to be involved in the cortical localisation of Mid1, which in turn specifies the division site and initiates CAR assembly [7]. In agreement with the role of Nod1 in this process, we found that septa in *nod1Δ* were slightly offset, a phenotype that was strikingly worsened in a *plo1* mutant background. It is possible that Nod1 interacts directly with Mid1 as part of the complex that it forms with Gef2. Intriguingly, we found that in the absence of *gef2*, Nod1 localised to the nucleus. To our knowledge, Nod1 is the only protein of the Wee1 pathway shown to behave in such a Mid1-like manner [5]. Further work would be required to determine if the nuclear localisation of Nod1 in *gef2Δ* cells is cell-cycle regulated, in a manner similar to Mid1 [37]. We surprisingly never observed a nuclear localisation for Gef2, even in the absence of Nod1. We envision that a role of Gef2 may be to sequester Nod1 away from the nucleus and Mid1. This would make Gef2 a negative regulator of Nod1 nuclear localisation.

### Un-coupled Node-containing Proteins and Actomyosin Rings

Nodes are clusters of many different proteins, which are believed to be the precursors of the actomyosin ring. This model is based on observations made by several groups that: 1) the interphase nodes recruit the actomyosin machinery. Indeed, nodes are placed at the cell midzone prior to actin and when actin is recruited at the onset of mitosis, it co-localises with the nodes. 2) the actomyosin machinery brings the nodes together by a search and capture mechanism. The rings consisting of node proteins and the actin ring are presumably intertwined because they co-localise throughout formation and constriction. As such, it is admitted that there is only one cytokinetic ring.

We describe for the first time a situation where the recruitment, constriction and disassembly of the rings made of node-containing proteins is uncoupled from that of the actomyosin machinery. For example, in *gef2Δ* cells, Blt1 and Nod1 assembled into a ring after Rlc1. Our data suggests that the actomyosin ring and the rings formed of node proteins appear as different structures. In fact, Nod1-tdTomato and Blt1-GFP, although not capable to condense into a proper ring *per se*, organised as filaments despite the absence of the functional actin nucleating formin Cdc12. This is in good agreement with previously published observations that the localisation of node-containing proteins is insensitive to the actin poison Latrunculin-A [7], [13]. Our result suggests that Nod1 and Blt1 are capable of “polymerising” without any pulling force provided by the myosins. The fact that these spring-like structures could not arrange as a ring in the *cdc12-112* strain however supports the idea that actin is essential for this process. Yet, we found that the integrity of the Blt1 and Nod1 rings could be compromised independently of the CAR. We believe that several rings exist during cytokinesis, which are anchored to the actomyosin ring either directly or *via* other intermediate rings. The function of some of these rings, such as that made of Blt1 that we placed beneath the cell wall, may be to bridge the plasma membrane with the actomyosin ring [36]. Gef2 may constitute an intermediate linker ring because in its absence (either by deletion or because it is not recruited in the *nod1Δ* strain), the late Nod1-ring collapses and the Blt1 ring scatters as a non-contractile disk. This would be in good agreement with our immunoprecipitation data, which showed that the Nod1-Blt1 interaction is mediated by Gef2. The reason for the existence of several rings at the cell equator is at present unclear and remains to be determined.

## Supporting Information

Figure S1 **Expression levels of Nod1-3PK in mutants of the nodes pathway.** Whole cell extracts of asynchronous cultures were loaded on a denaturing gel and Nod1-3PK was immunodetected with an anti 3PK antibody. Band intensities were measured using Photoshop and normalised to the α-tubulin loading control. The level of Nod1-3PK is decreased in *blt1Δ* and *gef2Δ* cells.
(PDF)

Figure S2 **Differential recruitment of Blt1-GFP in various strains.** Time-lapse imaging of Blt1-GFP in wild-type and *nod1Δ* cells. Rlc1-mCherry was used as an actomyosin marker and time 0 corresponds to the first recruitment of Rlc1 in cytokinesis nodes. Orange lines mark the site of future ring assembly, in the middle of the belt of nodes in the wild-type, and at its edge in *nod1Δ* cells. Orange arrows point at the cell tips, to which Blt1-GFP localise in the *nod1Δ* cell (see also Figure 4A). Bar = 5 µm.
(PDF)

Figure S3 **Nod1 and Blt1 form spirals in the absence of actin nucleation.** (A) Field of three individual *gef2Δ*cells pictured together. Transversal views of rings show three different stages of constriction. Cells expressing Blt1-GFP (green) and Nod1-tdTomato (red) were stained with calcofluor (blue) and combinations of merged images are shown. The cell wall encapsulates the Blt1 disk, which itself encloses the Nod1 ring. Bar = 2 µm. (B) Fields of *cdc12-112* cells expressing Blt1-GFP and Rlc1-mCherry observed at the permissive and restrictive temperature. Bar = 5 µm.
(PDF)

Table S1 **Fission yeast strains used in this study is listed.**
(PDF)

Movie S1 ***gef2Δ*** **cells form offset rings.** Time-lapse imaging of Blt1-GFP in wild-type (left) and *gef2Δ* (right) cells. In wild-type cells the ring assembles in the middle of the belt, whereas in *gef2Δ*cells it forms at its edge. Selected examples are shown with a line marking the width of the belt of nodes and arrows pointing at the site of ring assembly. Note that in *gef2Δ*cells the Blt1-GFP ring does not constrict. Time increment: 5 min per frame. Bar = 5 µm.
(MOV)
