# Supplementary figures and images for: Fission Yeast Nod1 Is a Component of Cortical Nodes Involved in Cell Size Control and Division Site Placement
Source: PLoS One. 2013 Jan 17;8(1):e54142. doi: 10.1371/journal.pone.0054142 (PMC3547912; doi:10.1371/journal.pone.0054142)

Supplementary Figure S1: Jourdain *et al.*

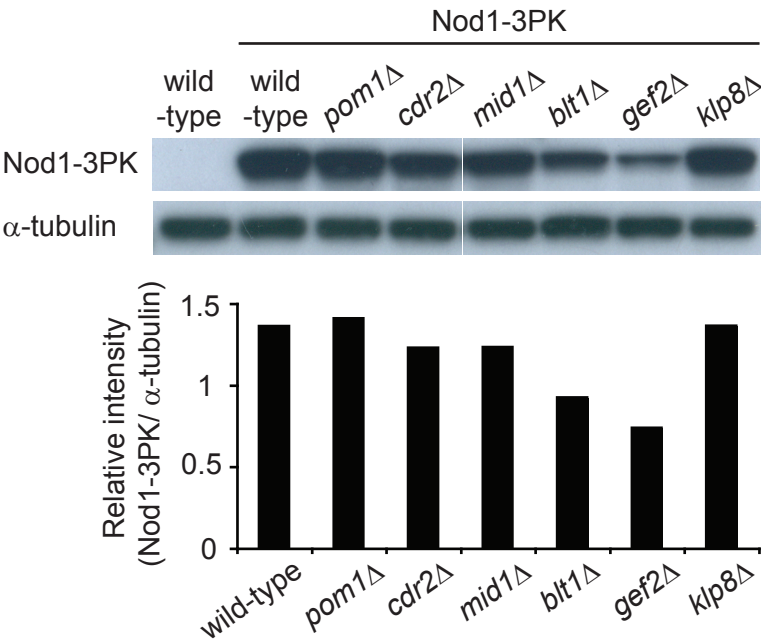

Supplement: Figure S1 — Expression levels of Nod1-3PK in mutants of the nodes pathway. Whole cell extracts of asynchronous cultures were loaded on a denaturing gel and Nod1-3PK was immunodetected with an anti 3PK antibody. Band intensities were measured using Photoshop and normalised to the α-tubulin loading control. The level of Nod1-3PK is decreased in blt1Δ and gef2Δ cells. (PDF) [file pone.0054142.s001.pdf]

Supplementary Figure S2: Jourdain *et al.*

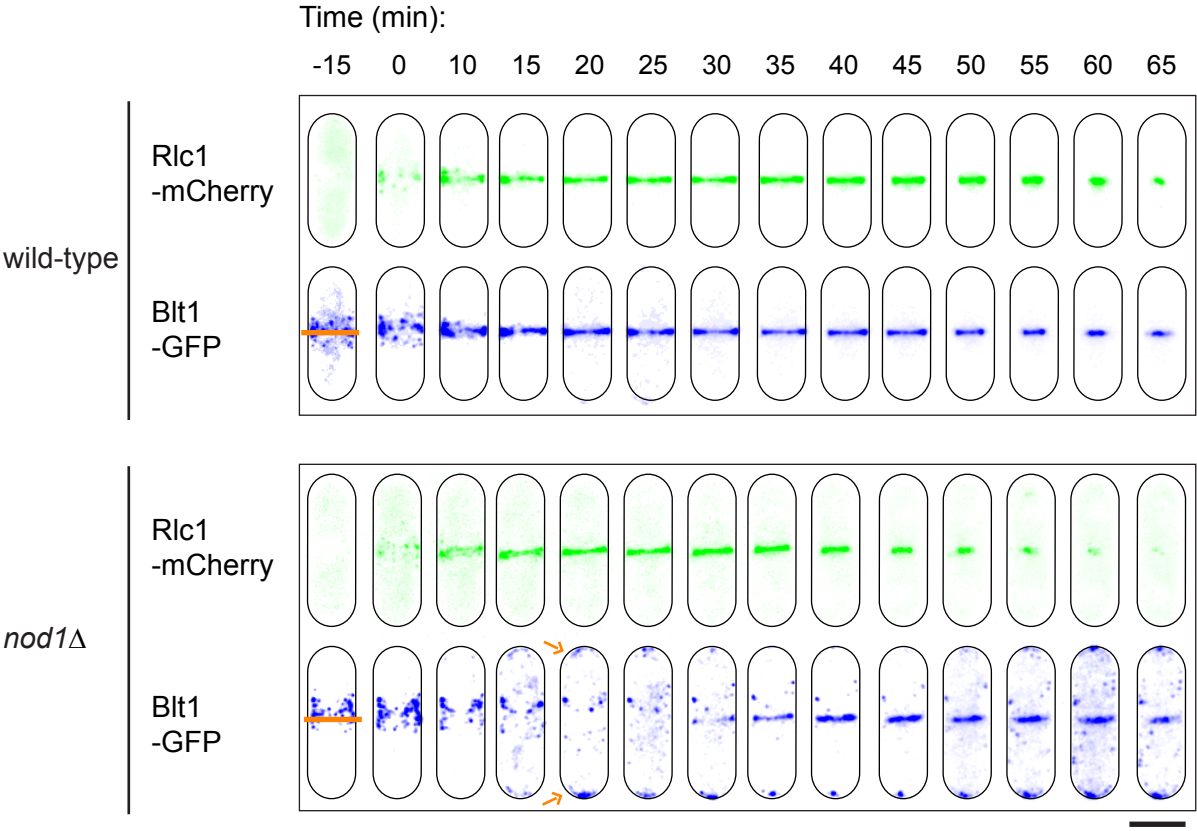

Supplement: Figure S2 — Differential recruitment of Blt1-GFP in various strains. Time-lapse imaging of Blt1-GFP in wild-type and nod1Δ cells. Rlc1-mCherry was used as an actomyosin marker and time 0 corresponds to the first recruitment of Rlc1 in cytokinesis nodes. Orange lines mark the site of future ring assembly, in the middle of the belt of nodes in the wild-type, and at its edge in nod1Δ cells. Orange arrows point at the cell tips, to which Blt1-GFP localise in the nod1Δ cell (see also Figure 4A). Bar = 5 µm. (PDF) [file pone.0054142.s002.pdf]

# Supplementary Figure S3

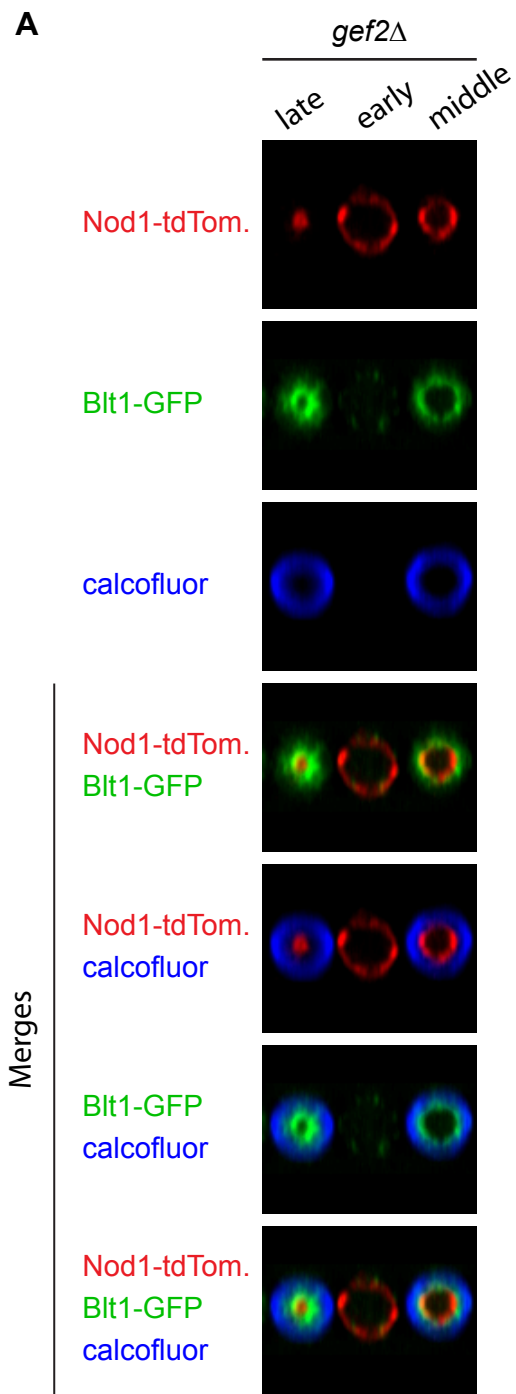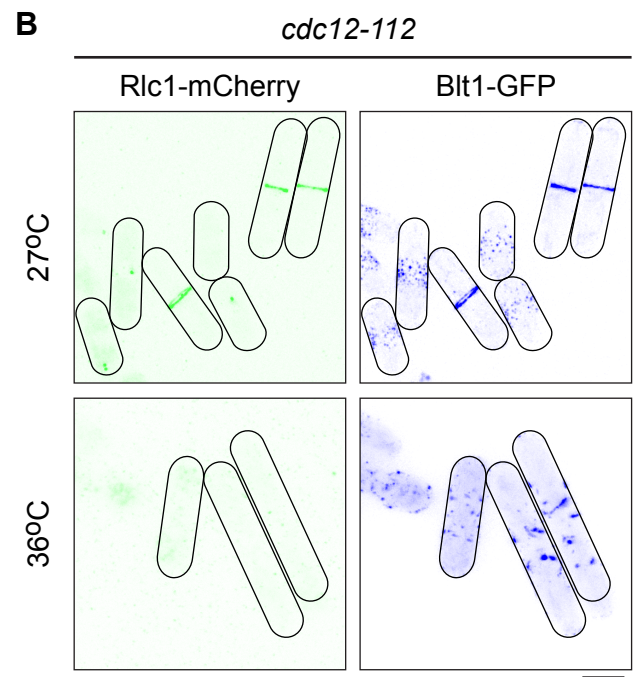

Supplement: Figure S3 — Nod1 and Blt1 form spirals in the absence of actin nucleation. (A) Field of three individual gef2Δcells pictured together. Transversal views of rings show three different stages of constriction. Cells expressing Blt1-GFP (green) and Nod1-tdTomato (red) were stained with calcofluor (blue) and combinations of merged images are shown. The cell wall encapsulates the Blt1 disk, which itself encloses the Nod1 ring. Bar = 2 µm. (B) Fields of cdc12-112 cells expressing Blt1-GFP and Rlc1-mCherry observed at the permissive and restrictive temperature. Bar = 5 µm. (PDF) [file pone.0054142.s003.pdf]
