# Supplementary material for: Fission Yeast Nod1 Is a Component of Cortical Nodes Involved in Cell Size Control and Division Site Placement
Source: PLoS One. 2013 Jan 17;8(1):e54142. doi: 10.1371/journal.pone.0054142 (PMC3547912; doi:10.1371/journal.pone.0054142)
Supplement: Table S1 — Fission yeast strains used in this study is listed. (PDF) [file pone.0054142.s004.pdf]

Supplementary Table S1: *S. pombe* strains used in this study

a : auxotrophy
h : mating type
ND : not determined

Strains	Genotype	Source
IJ137	*ade6.M216 leu1.32 ura4-D18* h-	Lab stock
IJ640	*nod1::hph^R leu1.32* h-	This study
JHS335	*pom1::ura4$^+$ leu1.32 ura4-D18* h+	Lab stock
JM569	*cdr2::nat^R* h-	P. Nurse
JM555	*cdr1-kan^R h-*	P. Nurse
IJ525	*wee1-50 leu1.32* h-	Lab stock
IJ50	*mid1::ura$^+$ ade-? leu1-32 ura4-D18* h+	Lab stock
JM206	*blt1::kan^R* h+	P. Nurse
IJ610	*gef2::kan^R ade6.M216, leu1.32, ura4-D1*8 h+	This study
IJ915	*gef2::nat^R ade6.M216, leu1.32, ura4-D1*8 h+	This study
IJ608	*klp8::kan^R leu1-32 ura4-D18* h-	This study
IJ49	*cd12.112 leu1-32* h-	Lab stock
IJ705	*nod1::hph^R cdr2::nat^R*, a=ND h=ND	This study
IJ527	*nod1-tdTomato-hph*^{R} *ade6-M216 leu1.32 ura4-D18* h+	This study
IJ486	*nod1-gfp-kan*^{R}, *leu1.32, ura4-D18,* h-	This study
IJ504	*nod1-gfp-kan*^{R} *pom1::ura4$^+$* a=ND h=ND	This study
IJ550	*nod1-tdTomato-hph*^{R} *cdr2::nat^R* a=ND h=ND	This study
IJ552	*nod1-tdTomato-hph*^{R} *cdr1::kan^R* a=ND h=ND	This study
IJ567	*nod1-tdTomato-hph*^{R} *blt1::kan^R* a=ND h=ND	This study
IJ558	*nod1-tdTomato-hph*^{R} *wee1-50* a=ND h=ND	This study
IJ562	*nod1-tdTomato-hph*^{R} *mid1::ura$^+$* a=ND h=ND	This study
IJ568	*nod1-tdTomato-hph*^{R} *blt1::kan^R* a=ND h+	This study
IJ625	*nod1-tdTomato-hph*^{R} *gef2::kan^R* a=ND h+	This study
IJ617	*nod1-tdTomato-hph*^{R} *klp8::kan^R* a=ND h=ND	This study
IJ535	*nod1-tdTomato-hph*^{R} *cdc12.112 leu1.32* h-	This study
JM151	*blt1-egfp-kan^R ade6-M21? leu1-32, ura4-D18* h-	P. Nurse
IJ917	*blt1-egfp-nat^R ade6-M? leu1-32 ura4-D18,* h-	This study
IJ556	*blt1-egfp-kan^R nod1-tdTomato-hph*^{R} a=ND h=ND	This study
IJ586	*blt1-egfp-kan^R nod1::hph^R ade6-M? leu1-32 ura4-D18* h=ND	This study
IJ927	*blt1-egfp-nat^R gef2::kan^R* a=ND h=ND h-	This study
IJ933	*blt1-egfp-nat^R nod1-tdTomato-hph*^{R} *gef2::kan^R* a=ND h=ND	This study
IJ947	*blt1-egfp-kan^R cdc12.112* a=ND h+	This study
IJ783	*gef2-3gfp-nat^R leu-* h-	This study
IJ979	*gef2-3gfp-nat^R blt1::kan^R* a=ND h=ND	This study

IJ951	*gef2-3gfp-nat*^{R} *nod1::hph*^{R} a=ND h+	This study
IJ774	*nod1-3pk-hph*^{R} *leu1-32* h-	This study
IJ875	*nod1-3pk-hph*^{R} *pom1::ura4*$^{+}$ a=ND h=ND	This study
IJ835	*nod1-3pk-hph*^{R} *cdr2::nat*^{R} a=ND h=ND	This study
IJ877	*nod1-3pk-hph*^{R} *mid1::ura4*$^{+}$ a=ND h=ND	This study
IJ781	*nod1-3pk-hph*^{R} *blt1::kan*^{R} a=ND h+	This study
IJ801	*nod1-3pk-hph*^{R} *gef2::kan*^{R} a=ND h-	This study
IJ837	*nod1-3pk-hph*^{R} *klp8::kan*^{R} a=ND h=ND	This study
IJ910	*blt1-3Flag-kan*^{R} *ade6.M216 leu1-32 ura4-D18* h-	This study
IJ931	*nod1-3pk-hph*^{R} *blt1-3Flag-kan*^{R} a=ND h-	This study
IJ980	*nod1-3pk-hph*^{R} *blt1-3Flag-kan*^{R} *gef2::nat*^{R} a=ND h=ND	This study
IJ929	*nod1-3pk-hph*^{R} *gef2-3gfp-nat*^{R} a=ND h+	This study
IJ977	*nod1-3pk-hph*^{R} *gef2-3gfp-nat*^{R} *blt1::kan*^{R} a=ND h=ND	This study
IJ975	*blt1-3Flag-kan*^{R} *gef2-3gfp-nat*^{R} a=ND h=ND	This study
IJ983	*blt1-3Flag-kan*^{R} *gef2-3gfp-nat*^{R} *nod1::hph*^{R} a=ND h=ND	This study
IJ945	*nod1-3pk-hph*^{R} *blt1-3Flag-kan*^{R} *gef2-3gfp-nat*^{R} a=ND h=ND	This study
SO3516	*rlc1-mcherry-ura*$^{+}$ *ade6-M? leu1-32 ura4-D18,* h-	Lab stock
SO892	*rlc1-gfp-ura*$^{+}$ *leu1-32 ura4-D18,* h+	Lab stock
IJ986	*rlc1-mcherry-ura*$^{+}$ *blt1-egfp-kan*^{R} a=ND h=ND	This study
IJ985	*rlc1-mcherry-ura*$^{+}$ *blt1-egfp-nat*^{R} *gef2::kan*^{R} a=ND h=ND	This study
IJ988	*rlc1-gfp-ura*$^{+}$ *nod1-tdTomato-hph*^{R} a=ND h=ND	This study
IJ987	*rlc1-gfp-ura*$^{+}$ *nod1-tdTomato-hph*^{R} *gef2::nat*^{R} a=ND h=ND	This study
IJ1168	*rlc1-mcherry-ura*$^{+}$ *blt1-egfp-nat*^{R} *nod1::hph*^{R} a=ND h=ND	This study
